# Brazilian guidelines for the monitoring and treatment of pediatric allergic conjunctivitis

**DOI:** 10.5935/0004-2749.20220053

**Published:** 2025-08-21

**Authors:** Cristiana Soares Ronconi, Dayane C. Issaho, Fabio Ejzenbaum, Luisa Moreira Hopker, Dirceu Solé, Herberto J. Chong-Neto, Ana Tereza R. Moreira, Marcia Keiko Tabuse, Myrna S. Santos, Julia Dutra Rossetto

**Affiliations:** 1 Ophthalmology and Visual Science Department, Universidade Federal de São Paulo, São Paulo, SP, Brazil; 2 Ophthalmology Department, Hospital de Olhos do Paraná, Curitiba, PR, Brazil; 3 Ophthalmology Department, Santa Casa de Misericórdia de São Paulo, São Paulo, SP, Brazil; 4 Pediatrics Department, Universidade Federal de São Paulo, São Paulo, SP, Brazil; 5 Pediatrics Department, Universidade Federal do Paraná, Curitiba, PR, Brazil; 6 Department of Ophthalmology and Otorhinolaryngology, Universidade Federal do Paraná, Curitiba, PR, Brazil; 7 Pediatric Ophthalmology Department, Instituto de Puericultura e Pediatria Martagão Gesteira, Universidade Federal do Rio de Janeiro, Rio de Janeiro, RJ, Brazil

**Keywords:** Conjunctivitis, allergic, Rhinitis, allergic, seasonal, Hypersensitivity, Child, Conjuntivite alérgica, Rinite alérgica sazonal, Hipersensibilidade, Criança

## Abstract

Allergic conjunctivitis is an increasingly frequent condition with a higher prevalence in children. It can be debilitating and is responsible for a great economic burden. These guidelines were developed on the basis of the medical literature (PubMed/Medline database) and the experience of an Expert Committee composed of members of the Brazilian Society of Pediatric Ophthalmology, the Brazilian Council of Ophthalmology, the Brazilian Society of Pediatrics, and the Brazilian Association of Allergy and Immunology. Allergic conjunctivitis is considered to be controlled when the ocular symptoms are not uncomfortable or are present, at most, on 2 days a week; the visual analog scale score is below 5; and the degree of conjunctival hyperemia is graded 0 or 1 on the Efron scale. Allergic conjunctivitis should be classified as mild, moderate, severe, and vision-threatening for adequate treatment and monitoring of frequency. The present document is a guideline for diagnosing, treating, and monitoring pediatric allergic conjunctivitis considering the clinical and demographic aspects of allergic conditions in Brazil.

## Abstract (pt)

A conjuntivite alérgica (CA) é uma condição frequente, debilitante e responsável por grande impacto econômico, proporcionalmente maior quando acomete crianças. Essas diretrizes foram desenvolvidas com base na literatura científica (PubMed/Medline) e na experiência de um Comitê de Especialistas composto por membros da Sociedade Brasileira de Oftalmologia Pediátrica, do Conselho Brasileiro de Oftalmologia, da Sociedade Brasileira de Pediatria e da Associação Brasileira de Alergia e Imunologia. A conjuntivite alérgica é considerada controlada quando os sintomas não são desconfortáveis ou estão presentes por dois dias na semana; o escore visual pela escala analógica é inferior a 5 e o grau de hiperemia conjuntival é de 0-1 pela escala de Efron. A conjuntivite alérgica deve ser classificada em leve, moderada, grave e com risco de perda visual para tratamento e frequência de monitoramento adequados. Esta diretriz orienta o diagnóstico, tratamento e monitoramento da conjuntivite alérgica pediátrica, considerando aspectos clínicos e demográficos das condições alérgicas no Brasil.

## INTRODUCTION

Allergic conditions affect 30% to 50% of the world population, and ocular symptoms are present in 40% to 60% of affected individuals^(1,2)^. The prevalence of allergic conditions is showing consistent increases, probably related to genetic predisposition combined with environmental factors (e.g., food, allergens, and pollution)^(2)^.

Brazilian data report a prevalence of rhinoconjunctivitis of 15% to 28%^(3,4)^. Up to 44% of asthmatic children under 14 years of age report at least one eye symptom, although only a third of them have a medical diagnosis of allergic conjunctivitis (AC)^(5)^.

Currently, no guidelines have been established for monitoring and treating AC in children and adolescents in Brazil. This is a frequent underdiagnosed condition that has an impact on quality of life and serves as a trigger for ocular complications. The objective of this document is to guide the monitoring and treatment of AC in children and adolescents in Brazil.

## METHODS

These guidelines focused on scientific rigor, clinical applicability, and editorial independence and sought clarity on communicating the recommendations. They were developed on the basis of careful consideration of the medical literature and the clinical experience of the Expert Committee of the Brazilian Pediatric Ophthalmology Society (SBOP) to define the optimal management of pediatric AC.

For that purpose, a literature review of symptoms, diagnosis, and treatment of AC was carried out in PubMed database, up to 2019, using the following terms: ocular allergy OR (classification AND AC) OR (diagnosis AND AC) OR (differential diagnosis AND ocular allergy) OR (treatment AND AC) OR (quality of life AND allergic diseases) OR (systemic immunosuppression AND AC) OR (control AND allergic diseases).

A group of experts composed of members of the SBOP, the Brazilian Council of Ophthalmology (CBO), the Brazilian Society of Pediatrics (SBP), and the Brazilian Association of Allergy and Immunology (ASBAI) reviewed the data and selected 56 scientific studies, including meta-analyses, systematic reviews, randomized controlled trials (RCTs), case-control studies, observational studies, and case reports of AC, based on the following levels of evidence^(6)^:

**1++** High-quality meta-analyses, systematic reviews of RCTs, or RCTs with a very low risk of bias;
**1+** Well-conducted meta-analyses, systematic reviews of RCTs, or RCTs with a low risk of bias;
**1-** Meta-analyses, systematic reviews or RCTs, or RCTs with a high risk of bias;
**2++** High-quality systematic reviews of case-control or cohort studies, or high-quality case-control or cohort studies, with a very low risk of confounding bias;
**2+** Well-conducted case-control or cohort studies with a low risk of confounding bias;
**2-** Case-control or cohort studies with a high risk of confounding bias;
**3** Nonanalytic studies (case reports, case series);
**4** Expert opinion.

The recommendations suitable to the target population were classified according to the scale of the Scottish Intercollegiate Guidelines Network, as described below^(6)^.

- At least one meta-analysis, systematic review, or RCT rated as 1++, or a systematic review of RCTs, or a body of evidence consisting principally of studies rated as 1+ and demonstrating overall consistency of results;
- Evidence including studies rated as 2++ and de-monstrating overall consistency of results, or extrapolated evidence from studies rated as 1++ or 1+;
- Evidence including studies rated as 2+ and demons-trating overall consistency of results, or extrapolated evidence from studies rated as 2++;
- Evidence level 3 or 4, or extrapolated evidence from studies rated as 2+.

For issues without scientific evidence, the proposals were based on expert consensus.

All the entities involved approved the final guideline document. Institutional review board approval was not obtained, since this report does not contain any original data or personal information that could lead to the identification of patients.

## RESULTS AND RECOMMENDATIONS

### Pathophysiology

The pathophysiology of acute allergic reaction in the conjunctiva is predominantly due to inflammation that depends on immunoglobulin E (IgE). Chronic ocular aller gy involves the activity of inflammatory cells (eosinophils, T lymphocytes) and the production of cytokines. The allergic process occurs basically in two stages^(2)^. The first stage involves the activation of Langerhans cells, which interact and present the antigen to the helper T lymphocytes. The helper T lymphocytes produce interleukins (ILs) that stimulate B-lymphocytes, diverting the production of the specific allergen IgG to IgE. In the second stage, the specific allergen IgE attaches to the mast cells and/or basophils by their surface high-affinity receptors. The interaction between the allergen and this specific IgE determines the degranulation of mast cells with the accompanying production and release of inflammatory mediators, such as vasoactive mediators (histamine), stored in their intracellular granules^(7)^.

### Classification Of AC

AC is classified into six ocular types: seasonal AC, perennial AC, atopic keratoconjunctivitis, vernal keratoconjunctivitis, giant papillary conjunctivitis, and allergic contact conjunctivitis^(2,8-10)^.

1. **Seasonal and perennial allergic conjunctivitis (SAC, PAC):** SAC is the most prevalent form of ocular allergy, affecting 22% of the population^(2)^. Its symptoms appear seasonally and last for less than 4 weeks. In contrast, PAC is characterized by signs and symptoms that persist for 4 days a week and for more than four consecutive weeks^(2)^. Patients present with pruritus, hyperemia, papillary conjunctival reaction, tearing, and eyelid edema. Chemosis and serous to mucous discharge may be present.
2. **Atopic keratoconjunctivitis (AKC): AKC** is generally severe and chronic and affects mostly men from the third to the fifth decades of life. It is associated with atopic dermatitis in almost 100% of cases^(2)^. Typical findings are gelatinous hyperplasia of the limbus, Horner- Trantas nodules, and prominent papillary hypertrophy in the lower tarsal conjunctiva. Severe cases present with scars, eyelid retraction, and loss of eyelashes and may result in reduced visual acuity due to epithelial defects, limbal deficiency, and corneal opacity.
3. **Vernal keratoconjunctivitis (VKC): VKC** is a rare and severe form of eye allergy that occurs in the first decade of life in approximately 80% of patients, with a slight predominance in males. Associations with other atopic features occur in about 50% of cases^(2,8)^. In most cases, the disease is self-limiting, tending to resolve after puberty. Typical findings include giant papillae, Horner-Trantas nodules in the limbus, and shield ulcers. Central involvement of the cornea may also occur, with neovascularization and opacity.
4. **Giant papillary conjunctivitis (CPG): CPG** is induced by mechanical irritation from contact lenses, ocular prosthesis, or ocular sutures. It usually presents as proliferative changes in the upper eyelid conjunctiva.
5. **Allergic contact conjunctivitis (CAC): CAC** occurs after sensitization of the eye by contact, for example, with topical drugs.

### AC diagnosis

The diagnosis of ocular allergy is based on family and personal history of atopy, symptoms, clinical signs, and, eventually, additional tests^(11)^. Ocular allergy can be associated with allergic rhinitis in 97% of children, asthma in 56%, and atopic dermatitis in 33%^(12-15)^. It is usually bilateral, with itching, accompanied by tearing and a burning sensation, as the most common symptom. Visual disturbance and photophobia can occur in severe cases^(9)^.

A slit-lamp ophthalmological examination may reveal watery or mucoid secretions, eyelid edema, chemosis, papillary hypertrophy in the palpebral conjunctiva, conjunctival hyperemia, limbal nodules, keratitis, and corneal involvement^(1)^.

Complementary tests, such as skin tests, and measurement of IgE specific levels in serum or tears can be requested. However, skin tests tend to be negative in the absence of an association with rhinitis, and the IgE dosages may not be conclusive, since 24% of patients may be sensitive to multiple allergens^(2)^. Thus, cytological diagnosis is usually reserved for research purposes^(16)^.

### AC treatment

The initial treatment consists of nonpharmacological measures that aim to prevent or minimize contact between the allergen and the conjunctiva. If nonpharmacological measures are insufficient, topical pharmacological treatment is started with antihistamines, mast cell membrane stabilizing agents, multiple-action drugs, nonsteroidal anti-inflammatory drugs (NSAIDs), and corticosteroids (Table 1)^(17)^. Systemic allergen-specific immunotherapy can be used to suppress or regulate the immune response. Immunotherapy not only helps to control symptoms but also slows the progression of the allergic disease.

**Table 1:** Medication class, mechanisms of action, side effects, and dosage of topical treatment of allergic conjunctivitis

Classification		Mechanism of action	Side effects	Topical ophthalmic agents generic name	Dosage	
Artificial tears		Dilution and removal of antigens from the eye surface	Chronic use can lead to chemical conjunctivitis due to preservative exposure	Cellulose derivative	With preservatives: 1 drop up to 6 times daily Preservative free: unlimited use	
Topical decongestants		Vasoconstriction via stimulation of alpha-adenoreceptors	Chemical conjunctivitis, follicular reaction, rebound hyperemia, pupillary dilation; contraindicated in patients with narrow-angle glaucoma	Emedastine, ephedrine, naphazoline, pheniramine	1 or 2 drops up to 4 times daily	
Topical antihistamines		Relatively selective histamine receptor antagonist	Ocular burning, headache, bitter taste	Azelastine, levocabastine	1 or 2 drops up to 4 times daily	
Mast cell stabilizers		Mast cell degranulation blockage, stabilizing the cell and preventing the release of histamine and related mediators	Ocular burning, stinging and itching sensations	Sodium chromoglycate, lodoxamide tromethamine, nedocromil	1 or 2 drops twice up to 4 times daily	
Multiple-action agents		Selective H1-receptor antagonists and mast cell stabilizers	Itching, irritation, burning, stinging sensations, eye redness	Olopatadine, alcaftadine, ketotifen	1 drop once daily 1 drop up to 3 times daily	
NSAIDs		Cyclooxygenase and prostaglandins blockage	Ocular burning, stinging, and itching sensations	Ketorolac tromethamine, diclofenac, nevanac	1 drop up to 6 times daily	
Corticosteroids		Interfere with intracellular protein synthesis and cause blockage of phospholipase A2, the enzyme responsible for the formation of arachidonic acid	Intraocular pressure increase, cataracts	Loteprednol, prednisolone, fluormetolone, dexametasone	2/2 hs-4/4 hs for 3-4 weeks Taper when using for more than 7 days	
	Immunosuppressors	Anti-inflammatory/immunomodulatory activity by inhibiting the activation of NF-kB, a nuclear factor involved in regulation of immune and proinflammatory cytokine genes	Eye burning, headache, foreign body sensation, conjunctival hyperemia	Cyclosporin Tacrolimus	1%-2%: 2-4 times daily 0.05%: 2-4 times daily Ointment 0.02%-0.03% Drops 0.03%-0.1% 2-4 times daily	

NF-kB= nuclear factor kB; NSAIDS= nonsteroidal anti-inflammatory drugs.

#### Nonpharmacological measures

Nonpharmacological measures include general environmental measures to reduce exposure to allergens (e.g., elimination of domestic dust, fungi, and pollen) and specific actions, such as the use of cold-water compresses, preservative-free artificial tears, and local cleansing with saline solution to wash the allergens from the conjunctiva and to contract the conjunctival vessels to relieve edema and hyperemia^(10)^. In addition, sunglasses can be used to prevent contact with suspended allergens and for photophobia relief (grade of recommendation A)^(18)^.

#### Topical treatment (Table 1)

- First-generation topical eye antihistamines act by blocking receptor H1; however, they are poorly tolerated and have limited effect and potency (grade of recommendation D)^(7,19)^. Their combination with vasoconstrictors extends their therapeutic effect, although at the expense of rebound hyperemia and tachyphylaxis as undesirable common adverse effects. Long-term use of vasoconstrictors is not recommended, and these drugs should be administered with caution to patients with glaucoma, hyperthyroidism, or cardiovascular disease (grade of recommendation D)^(20)^.
- Mast cell membrane stabilizers act by inhibiting mast cell degranulation^(19)^. These agents must be administered every 6 to 8 hours for at least 2 weeks; consequently, adherence to their use is generally low (grade of recommendation A)^(20)^.
- Multiple-acting agents act as mast cell stabilizers, selective H1 receptor antagonists (olopatadine and ketotifen), and modulators of the anti-inflammatory activity of eosinophils. Some, such as epinastine, act on H1 receptors (reducing itching) and H2 receptors (reducing vasodilation), whereas others, such as alcaftadine, also block H4 receptors. Multiple-acting agents have prompt and long-lasting effects and have proven more effective than fluorometholone in the treatment of SAC (grade of recommendation A)^(21,22)^.
- Topical NSAIDs act by blocking the cyclooxygenase pathway and thus the synthesis of prostaglandins and thromboxanes. These drugs have proven efficacy against hyperemia and conjunctival itching (grade of recommendation A)^(23)^. Ketorolac is approved for the treatment of AC but has been reported to be less effective than olopatadine and emedastine^(24)^. The use of topical NSAIDs in children is also limited by their burning sensation^(15)^.
- Corticosteroids interfere with intracellular protein synthesis and block phospholipase A2, the enzyme responsible for the formation of arachidonic acid. These drugs also act by inhibiting the production of cytokines and the migration of inflammatory cells. Topical ocular corticosteroids are not considered a first-choice therapy for AC, although drugs in lower concentrations (fluorometholone, loteprednol, and rimexolone) can be used to treat moderate inflammation^(15)^. The drugs of choice for severe inflammation are dexamethasone and prednisolone (grade of recommendation B)^(24)^ administered at high frequency (every 2 to 4 hours, depending on severity) for short periods (3 to 4 weeks). Potential adverse effects (e.g., increased intraocular pressure, cataracts, and keratitis) must be closely monitored. Patients with severe allergic keratoconjunctivitis, giant papillae, intense limbic involvement, or recurrent corneal ulcers can be given a supratarsal injection of corticosteroids as an adjuvant treatment option^(2)^. Satisfactory results can be achieved with injections of 0.4 to 0.5 mL of dexamethasone phosphate (4 mg/mL), prednisolone acetate (40 mg/mL), or triamcinolone acetate (10.5 mg/mL). Repeated injections may be indicated at intervals of approximately 6 months (grade of recommendation D).
- Topical nasal corticosteroids are not considered a treatment of choice for AC, but they can improve ocular symptoms by reducing the nasal-ocular reflex in patients with rhinoconjunctivitis. In particular, mometasone furoate^(25)^, fluticasone furoate^(26)^, and fluticasone plus azelastine furoate can alleviate the symptoms of allergic rhinoconjunctivitis (grade of recommendation A). Prolonged use over several months does not seem to generate a significant risk of increased intraocular pressure or glaucoma^(27)^. Regarding the effectiveness in controlling ocular symptoms in patients with allergic rhinoconjunctivitis, no superiority has yet been established between intranasal corticosteroids and oral antihistamines^(28)^.
- Immunomodulatory eye drops (cyclosporin and tacrolimus) are expected to have equivalent or better effects for long-term control than steroid eye drops and to spare their use^(29)^. Topical cyclosporin A exerts anti-inflammatory/immunomodulatory activity by inhibiting the activation of nuclear factor-kB (NF-kB), a nuclear factor involved in the regulation of cytokine genes of the immune and proinflammatory response. Cyclosporin A is available as 0.05% eye drops and is used 2 to 4 times a day^(29)^. If needed, a higher concentration of 1%-2% may be manipulated by specialized drugstores. Tacrolimus acts by inhibiting mast cell proliferation and degranulation and by reducing cytokine production by T lymphocytes through a mechanism similar to that of cyclosporin A, but with greater potency. It is available as ointment (0.02%0.03%) or eye drops (0.03%-0.1%) and is administered 2 to 4 times a day. It provides satisfactory results for improving symptoms, giant papillae, and corneal involvement^(30)^.

#### Systemic treatment

Systemic antihistamines compete with histamine for H1 receptors by inverse agonism, thereby blocking ocular symptoms, and especially the itching sensation, which depend on stimulation of H1 receptors. Some antihistamines are believed to exert anti-inflammatory effects by inhibiting the expression of intercellular adhesion molecules (ICAM-1) and affecting platelet activation factor (PAF) by significantly inhibiting PAF- induced mast cell activation.^(31,32)^. PAF is a lipid mediator involved in several allergic reactions, both in early and late phases. PAF is released from multiple cells of the immune system, such as eosinophils, neutrophils, and mast cells. First-generation H1 antihistamines are not recommended, because of their sedative effect and anticholinergic activity. Second-generation drugs (desloratadine, ebastine, loratadine, and rupatadine) have similar efficacy but a more manageable sedation profile and fewer adverse effects (grade of recommendation B)^(7)^. Antihistamine drugs are generally administered to control nasal and ocular symptoms in rhinoconjunctivitis^(10)^. However, dry keratoconjunctivitis has been linked to the use of oral antihistamines whose antimuscarinic activity causes abnormalities in the tear film^(33)^. These changes in the conjunctival epithelium may increase the inflammatory response to the allergen^(34)^.

#### Immunotherapy

High doses of allergens induce a deviation of the immune response in favor of Th1 lymphocytes, with the release of interferon gamma (IFN-γ) and production of regulatory T cells. The World Health Organization recommends specific immunotherapy for allergens as an effective approach in patients with allergic diseases, such as rhinoconjunctivitis and asthma^(30)^. Immunotherapy administered either sublingually or subcutaneously can induce tolerance to the allergen in the short and long term. The tolerance can be directed to Th1 responses by upregulating T-regulatory-cells secretion of the inhibitory cytokines IL-10 and/or by transforming growth factor β, which suppresses the allergen-specific Th2 response.^(30)^.

Specific immunotherapy improves ocular symptoms in patients with allergic rhinoconjunctivitis even after discontinuation of treatment^(30,35,36)^. The conjunctival sensitivity threshold to the allergen increases from before to after immunotherapy, and the treatment also produces a 63% reduction in the need for medication in patients with rhinoconjunctivitis or SAC, but not in patients with PAC (grade of recommendation A)^(37,38)^. The decision to start treatment depends on several factors: severity of the allergic disease, response to environmental prevention measures, patient acceptance, and adherence to treatment^(35)^.

#### Systemic immunosuppression

Immunomodulators may be an option for severe cases that are refractory to topical treatment to avoid systemic corticosteroid use and its inherent adverse effects^(39)^. Both tacrolimus and cyclosporin act by inhibiting calcineurin (a calcium-dependent phosphatase), which activates the nuclear factor and causes proliferation and activation of T cells. Inhibition of calcineurin activity restrains the second-messenger pathway involved in signal transduction, thereby inhibiting the production and activation of cytokines by T cells and, ultimately, the chronic inflammatory process. The use of T lymphocyte signal transduction inhibitors in the treatment of AKC in patients who are refractory to conventional therapy or other immunomodulatory therapy was shown to be effective in sparing the use of corticosteroids^(40)^. A minimum of 12 weeks of therapy with systemic cyclosporin at a daily dose of 3 to 5 mg/kg was required for a benefit in the treatment of atopic dermatitis^(41)^. The primary criterion for clinical diagnosis of AKC is demonstration of a specific form of chronic conjunctivitis and keratitis in association with atopic dermatitis or eczema^(42)^. That said, AKC is a systemic disease with local ocular manifestation^(43-45)^, and this is the reason for the use of systemic immunosuppression with cyclosporin A.

#### Monoclonal antibodies

Monoclonal antibody drugs are used to control allergic diseases and might also serve as an alternative treatment for eye allergies. The humanized anti-IgE monoclonal antibody omalizumab, which is indicated for the treatment of asthma and chronic urticaria, has also been shown to have an effect, although incomplete, on the control of severe VKC^(46)^. The anti-IL-4 medication dupilumab, which is indicated for atopic dermatitis, severe asthma, and chronic rhinosinusitis, might also be useful as an AC treatment, considering its mechanism of action.

#### Surgical treatment

Surgical treatment of AC includes superficial keratectomy procedures for shielded ulcer plaques, excision of giant papillae associated with autologous conjunctiva, oral mucosa or amniotic membrane grafts, and reconstructive surgery, such as limbal stem cell transplantation^(36)^.

Surgical intervention is reserved for patients with severe vision-threatening disease, characterized by the presence of large cobblestone papillae, active shield ulcers, limbic stem cell deficiency with extensive conjunctivalization, and extensive corneal scarring^(47)^. These patients are usually refractory to pharmacological therapy and need close monitoring for complications, such as infection, permanent corneal opacity, cataracts, and glaucoma^(47)^.

### Disease control criteria

**Figure 1:**
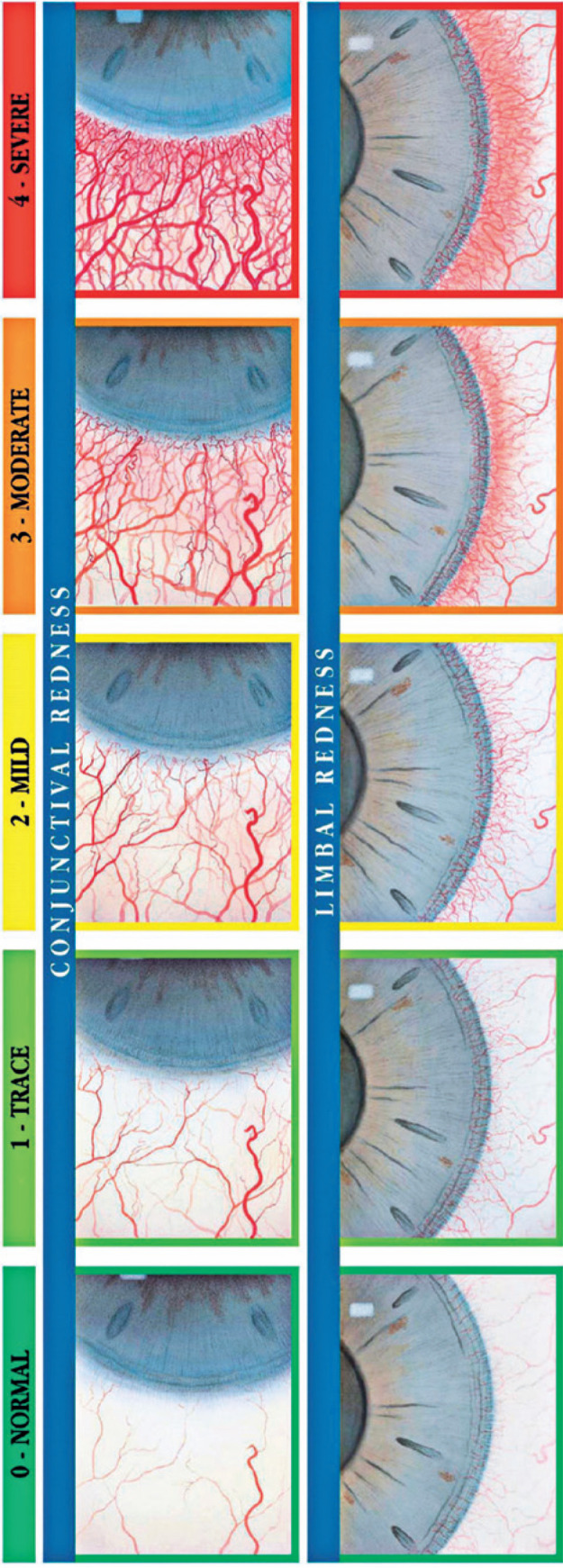
The Efron clinical grading scale for conjunctival and limbal hyperemia. Reprinted with permission from Efron N. Contact lens complications. 4th ed. Philadelphia: Elsevier; 2019 (ISBN: 978-0-7020-7611-4).

The present guidelines use the control criteria proposed by the Document on AC (DECA) (Table 2)^(15)^. The control criteria are based on the presence of ocular symptoms within 2 weeks of the evaluation, the visual analog scale (VAS) score, and the ophthalmological examination, with conjunctival hyperemia graded by the Efron scale (Figure 1)^(48)^.

**Table 2:** Clinical control criteria of allergic conjunctivitis

Symptoms	Controlled (all criteria below)	Uncontrolled (at least 1 of the following)
Itchiness Tearing Visual discomfort	No symptoms or symptoms <2 days/week	Any intensity if present >2 days/week
Visual Analog Scale	<5	>5
Hyperemia (Efron scale)	0-1	2-4

Control is achieved when the patient has no symptoms (itching, watery eyes, or visual discomfort) or when these symptoms are not uncomfortable or are present, at most, for 2 days a week^(15)^. Conversely, AC is considered uncontrolled if ocular symptoms, regardless of intensity, are present for more than 2 days a week and/or if their frequency and severity progress.

The VAS score has a good correlation with the symptom score and with the Rhinoconjunctivitis Quality of Life Questionnaire (using only the ocular symptom domain) ^(49,50)^. In the VAS assessment, the patients specify the point on the 0-10 scale that best corresponds to the severity of their symptoms. AC is considered controlled if the score is below 5^(49)^.

During the ophthalmological examination, control is assessed according to the degree of conjunctival hyperemia. AC is considered controlled if the degree of hyperemia on the Efron scale is 0 or 1 (Figure 1).

### Treatment algorithm

**Figure 2:**
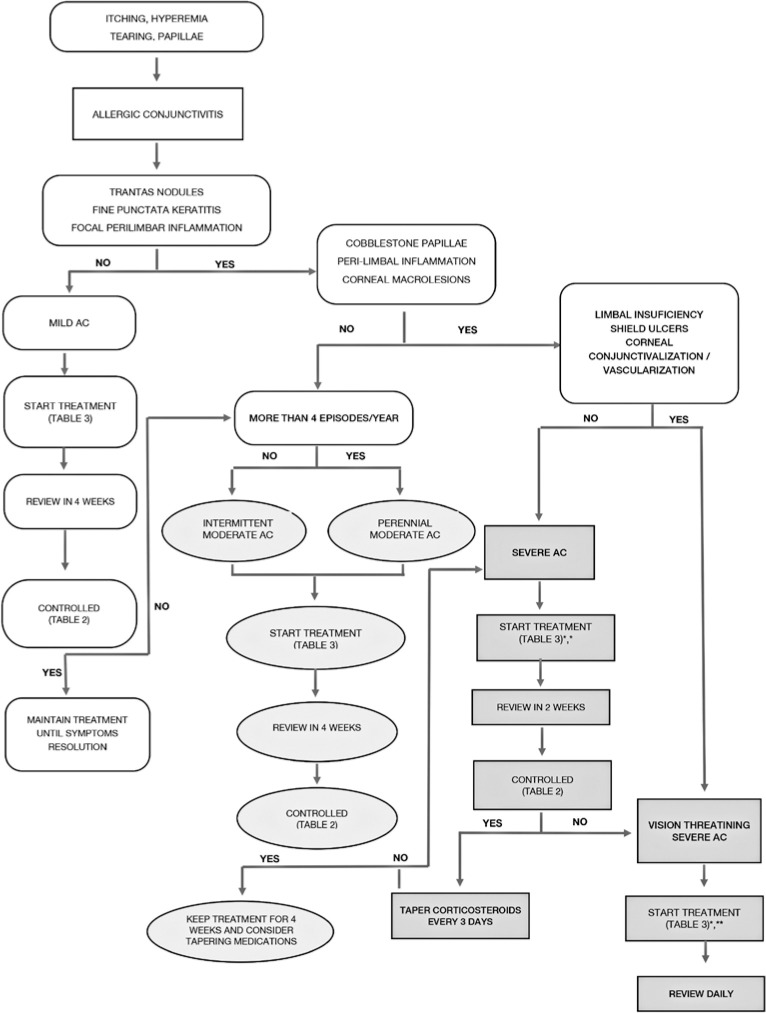
Algorithm for the classification and treatment of allergic conjunctivitis.

The present guidelines establish an algorithm for the classification and treatment of AC (Figure 2 and Table 3).

**Table 3:** Applicability of treatment modalities for allergic conjunctivitis according to its severity (grade of recommendation D)

Classification	Mild	Intermittent moderate	Perennial moderate	Severe	Vision-threatening conditions
Nonpharmacologic measures	**x**	**x**	**x**	**x**	**x**
Artificial tears	**x**	**x**	**x**	**x**	**x**
Topical decongestants	**x**	**x**	**x**	**x**	**x**
NSAIDs	**x**				
Selective H1-receptor antagonists and mast cell stabilizers	**x**	**x**	**x**	**x**	**x**
Mild corticosteroids		**x**	**x**		
Cyclosporin			**x**		
Corticosteroids		Only if corneal involvement	Only if corneal involvement	**x**	**x**
Tacrolimus				**x**	**x**
Supratarsal corticosteroids					**x**
Papillae excision					**x**
Ulcer debridement					**x**
Immunotherapy			x^*^	x^*^	x^*^
Monoclonal antibodies				x^**^	x^**^
Oral antihistamines/antileukotrienes		**x**	**x**	**x**	**x**
Nasal corticosteroids		x^***^	x^***^	x^***^	x^***^

NSAIDS= nonsteroidal anti-inflammatory drugs.

Consider specific immunotherapy in persistent cases or when associated with other allergies.

** Consider monoclonal antibody therapy.

*** When associated with nasal symptoms.

### Clinical monitoring

Ophthalmological assessments to assess AC control should be performed as follows:

**Mild Cases** - reassess every 4 weeks and maintain treatment until symptoms are resolved.

**Intermittent and Perennial Moderate Cases** - reassess every 4 weeks.

- Controlled: maintain treatment for 4 weeks and consider tapering the eye drops.
- Uncontrolled: treat as a severe case.
  OBS1: Consider using mild corticosteroids in cases of initial corneal involvement.
  OBS2: Consider specific immunotherapy in persistent cases or when associated with other manifestations of allergy.

**Severe Cases** - reassess every 2 weeks.

- Controlled: taper corticosteroid drops every 3 days. - Uncontrolled: reconsider the diagnosis.
  OBS1: Consider specific immunotherapy in persistent cases or when associated with other manifestations of allergy.
  OBS2: Consider therapy with biologicals.

## DISCUSSION

AC is an increasingly frequent condition that can be debilitating for the patient and challenging for the ophthalmologist. Genetic predisposition, combined with environmental exposure (food, allergens, viral infections, exposure to bacteria, use of NSAIDs, use of antibiotics, environmental pollution, etc.) may be responsible for the rising number of cases^(2)^. In most patients, AC is associated with other allergic conditions, especially rhinitis, justifying the term rhinoconjunctivitis. In children under 14 years of age, 44.7% of children with atopy had allergic rhinitis and 61% had conjunctivitis, but only 5% presented with conjunctivitis alone^(51)^. Ocular allergy presents with an obnoxious itching sensation, hyperemia, and tearing and can ultimately lead to corneal scarring and subsequent visual impairment. Approximately 11% of patients with AKC have persistent corneal epithelial defects^(1,2)^.

The Brazilian epidemiological data on ocular allergy in the general population indicate a prevalence of 17%^(8)^ for allergic rhinoconjunctivitis. Concerning the ocular allergy subtypes, a referral center reported that 38.7% of patients had VKC, 38.7% had AKC, 12.6% had PAC, and 10.1% had no definite diagnosis^(9)^. A predominance of chronic and severe forms of ocular allergy was also reported^(9)^. Among teenagers, the prevalence of rhinoconjunctivitis has been reported as ranging between 15% and 28%^(3,4)^. Among asthmatic children under 14 years of age, 44% reported at least one eye symptom, although only 16% had a medical diagnosis of AC; this indicates a high rate of underdiagnosis^(5)^.

Brazil is a continental country with a climate ranging from equatorial, in the north, to temperate, in the south. Some particularities may arise from this diversity, such as the type of allergen and the period of the year. Nevertheless, all the AC subtypes are covered in the present document, and, to our knowledge, there are no studies that suggest the need for regional adaptations. Therefore, these guidelines can be useful nationwide regardless of the region and climate.

AC, especially VKC, significantly affects the quality of life of patients and their families. It reduces their productivity^(2)^ and is an important component of the total economic burden of SAC, since it causes loss of time from work seeking medical attention and reduced productivity at work^(52)^. Reduction in productivity can be caused by SAC symptoms or by drowsiness caused by medications. Adults with SAC who used antihistamines reported a 25% reduction in productivity for an average of 14 working days per year^(52,53)^. By extrapolation, patients younger than 16 years with SAC may represent a proportionally greater economic burden, especially considering the impact of lower school attendance on future income-earning potential^(54)^.

AC treatment and monitoring are often challenging. The main goal of the treatment of ocular allergy is prompt reduction of inflammation to eliminate symptoms and to prevent complications such as dry eye and vision loss. Currently, different consensus exist worldwide for the treatment of AC ^(1,11,15,17,55)^. Guidelines are flexible tools based on the best scientific evidence and available clinical information, and they also reflect the consensus of experts in the field^(56)^. Genetic features and characteristics of exposure (geographic and climatic) are particularly pivotal in allergic disorders and must be considered when proposing a treatment guideline for a specific region.

After a thorough scientific review of AC, the aim of the SBOP in writing this consensus document was to establish guidelines for diagnosing, treating, and monitoring pediatric AC, considering the clinical and demographic aspects of allergic conditions in Brazil.
